# Identification of signature genes and immune infiltration analysis in thyroid cancer based on PANoptosis related genes

**DOI:** 10.3389/fendo.2024.1397794

**Published:** 2024-07-22

**Authors:** Yujie Li, Dengqiang Wu

**Affiliations:** ^1^ Department of Clinical Laboratory, The Affiliated LiHuiLi Hospital of Ningbo University, Ningbo, China; ^2^ Department of Clinical Laboratory, Ningbo No. 6 Hospital, Ningbo, China

**Keywords:** thyroid cancer, PANoptosis, immune infiltration, predictive models, candidate genes

## Abstract

**Background:**

Thyroid cancer is the most common malignancy of the endocrine system. PANoptosis is a specific form of inflammatory cell death. It mainly includes pyroptosis, apoptosis and necrotic apoptosis. There is increasing evidence that PANoptosis plays a crucial role in tumour development. However, no pathogenic mechanism associated with PANoptosis in thyroid cancer has been identified.

**Methods:**

Based on the currently identified PANoptosis genes, a dataset of thyroid cancer patients from the GEO database was analysed. To screen the common differentially expressed genes of thyroid cancer and PANoptosis. To analyse the functional characteristics of PANoptosis-related genes (PRGs) and screen key expression pathways. The prognostic model was established by LASSO regression and key genes were identified. The association between hub genes and immune cells was evaluated based on the CIBERSORT algorithm. Predictive models were validated by validation datasets, immunohistochemistry as well as drug-gene interactions were explored.

**Results:**

The results showed that eight key genes (NUAK2, TNFRSF10B, TNFRSF10C, TNFRSF12A, UNC5B, and PMAIP1) exhibited good diagnostic performance in differentiating between thyroid cancer patients and controls. These key genes were associated with macrophages, CD4+ T cells and neutrophils. In addition, PRGs were mainly enriched in the immunomodulatory pathway and TNF signalling pathway. The predictive performance of the model was confirmed in the validation dataset. The DGIdb database reveals 36 potential therapeutic target drugs for thyroid cancer.

**Conclusion:**

Our study suggests that PANoptosis may be involved in immune dysregulation in thyroid cancer by regulating macrophages, CD4+ T cells and activated T and B cells and TNF signalling pathways. This study suggests potential targets and mechanisms for thyroid cancer development.

## Introduction

Thyroid cancer (THCA) is one of the most common malignant tumours in the world, and in recent years, the incidence of thyroid cancer has been increasing every year. Compared with the total data in 2000, the incidence of thyroid cancer has increased 20 times (1, 2). The incidence of thyroid cancer in women is 3-4 times higher than in men. According to clinical and pathological typing thyroid cancer can be divided into papillary thyroid carcinoma (PTC), follicular thyroid carcinoma (FTC), medullary thyroid carcinoma (MTC), and undifferentiated thyroid carcinoma (ATC) (3, 4). PTC and FTC collectively referred to as differentiated thyroid carcinoma (DTC) is the most common pathological type of thyroid cancer, accounting for more than 90% of all thyroid cancers. The treatment of choice for most patients with thyroid cancer is surgical resection, and thyroid-stimulating hormone suppression and radioactive iodine (RAI) therapy are required for patients with high-risk features, with some patients with thyroid cancer progressing to RAI-refractory thyroid cancer and death (5). There is a lack of effective treatment strategies for patients with advanced thyroid cancer. The development, invasion, and metastasis of thyroid cancer are closely related to changes at the gene level and dysfunctional regulation of related signal transduction pathway. These molecular changes are the hallmarks of thyroid cancer diagnosis and prognosis, as well as potential targets for biological therapy.

PANoptosis is an inflammation-driven programmed cell death that combines key features of pyroptosis, apoptosis and necrotic apoptosis, yet cannot be characterised by any of these modes of death alone (6, 7). PANoptosis was first named in 2019 by the American scholar Malireddi, who proposed that the innate immune sensors ZBP1 and TAK1 kinase play important roles in the regulation of the PANoptosis vesicle complex (7). Three types of PANoptosis vesicles have been identified, ZBP1 PANoptosis vesicles, RIPK1 PANoptosis vesicles and AIM2 PANoptosis vesicles. Viruses, bacteria and other non-infectious factors such as cytokines in tumours can trigger host cells to undergo PANoptosis (8, 9). Pancytopenia is closely related to homeostasis maintenance, embryonic development, and immune regulation.

BRAF V600E and RAS gene variants are the most common mutations in thyroid cancer. These mutations constitutively activate the MAPK signalling pathway, and patients with advanced thyroid cancer develop other genetic variants in addition to these common mutations and become more aggressive and less differentiated (10, 11). The genetic variants and mechanisms that drive the development of thyroid cancer are becoming better understood, but researchers still do not fully understand the determinants and functional basis of certain genetic variants (12). The study of genomic profiles of thyroid cancer patients can help predict the prognosis of thyroid cancer patients and for subsequent immunotherapy. PANoptosis plays a crucial role in many diseases such as infections, tumours, and inflammatory diseases (13–16). There are no data from studies evaluating the impact of PANoptosis-related genes in THCA disease progression. Therefore, the present study proposes that PANoptosis-related genes may also be involved in thyroid cancer disease progression. We first analysed the expression levels of PANoptosis-related genes in THCA. The pathogenic mechanisms of PANoptosis-related genes were explored based on functional enrichment analysis. A PANoptosis risk score model was established by LASSO regression, and the diagnostic efficiency of key genes was verified in the validation dataset. Finally, we analysed the immune infiltration characteristics of THCA to reveal the association between key genes and the immune microenvironment. We aimed to explore the potential link between PANoptosis-related genes affecting the genetic variants of THCA, and to provide a theoretical basis for guiding the prognosis and immunotherapy of thyroid cancer patients.

## Materials and methods

### Data acquisition

The thyroid cancer datasets GSE33630 and GSE3467 were downloaded from the Gene Expression Omnibus (GEO) database (https://www.ncbi.nlm.nih.gov/gds). The training dataset GSE33630 consisted of 60 THCA patient samples and 45 control samples. Transcriptome information for 512 thyroid cancer samples was obtained from The Cancer Genome Atlas (TCGA) database (https://portal.gdc.cancer.gov/). The validation dataset GSE3467 included 9 THCA patient samples and 9 controls. The obtained dataset was analysed based on R software (version 4.3.1). PANoptosis-related genes were downloaded from the GeneCards database (https://www.genecards.org/).

### Analysis of PANoptosis related differentially expressed genes

We used the “combat” function in “sva” to remove the batch-to-batch difference to obtain the differentially expressed genes between the THCA group and the control group (17). The screening criteria were |logFC| > 1.5, p-value < 0.05. The obtained differentially expressed genes and PANoptosis related genes were imported into the jvenn online platform (https://jvenn.toulouse.inrae.fr/app/example.html) to obtain PANoptosis related differentially expressed genes (PRGs).

### Functional enrichment analysis

Differentially expressed genes were imported into the DAVID (https://david.ncifcrf.gov/) online platform for Gene Ontology (GO) and Kyoto Encyclopedia of Genes and Genomes (KEGG) analyses. GO mainly includes molecular function (MF), biological pathway (BP) and cellular component (CC). Visualization was performed using the “ggplot2” package in R software (18). The interaction network between PRGs was constructed using the STRING database. In addition, the metscape database (https://metascape.org/) was used to explore the functional mechanisms among PRGs.

### Screening for prognostic markers

Least Absolute Shrinkage and Selection Operator (LASSO) regression is a regularization method for linear regression problems, which can be used to reduce the complexity of the model, prevent overfitting, and select important feature variables (19). We used the LASSO regression model to screen for diagnostic markers. Patients with THCA in the training dataset were divided into high-risk and low-risk groups. Predictive model accuracy was assessed by area under the receiver operating characteristic curve (ROC). GSE3467 was used as a validation dataset to validate the expression of the key genes mentioned above and the area under ROC (AUC) value to measure the predictive power of the algorithm(P<0.05). We validated the predictive power of the algorithm in the Human Protein Atlas (HPA) database (https://www.proteinatlas.org/) to verify the expression levels of key genes in thyroid cancer tissues. The relationship between the core genes and the prognosis of thyroid cancer patients was explored by the clinical information in the TCGA database.

### Immune cell correlation analysis

The CIBERSORT algorithm was used to calculate the immune cell infiltration between the thyroid cancer group and the control group in the training dataset (20). Stacked plots were used to show the distribution of immune cells in each sample. Box plots show the relative proportions of the immune cell types between the two groups. The “ggcorrplot” package was used to explore the correlation between biomarkers and immune cell infiltration.

### Drug screening and predicting transcription factor regulatory networks

The Drug Gene Interaction Database (DGIbd) is a database for exploring drug-gene interactions. Transcription factors are key regulators of gene expression, and the activity of these proteins determines cellular function and response to environmental perturbations. We predicted TF regulatory networks and TF- miRNA regulatory networks of key genes by NetworkAnalyst 3.0 online tool (https://www.networkanalyst.ca/) (21). Analyses and presentations were performed using Cytoscape 3.7.2.

## Results

### Identification of differentially expressed genes of THCA

**Figure 1:**
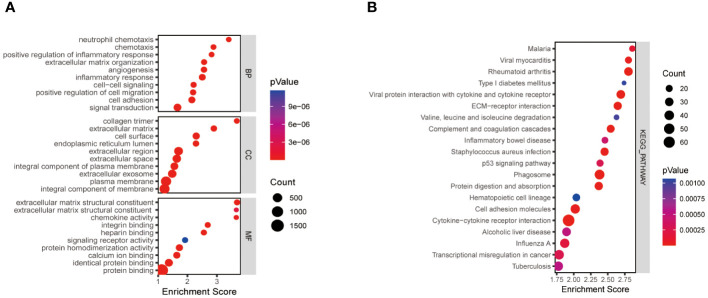
Functional enrichment analysis of thyroid cancer-related differentially expressed genes. The size of the ball represents the number of genes. The colours of the balls correspond to different P values. **(A)** The top 10 significantly enriched GO annotations, including biological process, cellular component, and molecular function, were selected separately. **(B)** The top 20 significantly enriched KEGG pathway analyses. GO, the Gene Ontology; KEGG, the Kyoto Encyclopedia of Genes and Genomes analyses.

We obtained a total of 2219 differentially expressed genes by analysing the training dataset GSE33630. The screening criteria were |logFC|>1.5, P>0.05. The volcano plots and heatmaps in **Supplementary Figures 1A, B** demonstrate the differential expression patterns of DEGs in the dataset GSE3360. These DEGs were mainly enriched in the positive regulation of inflammatory response, cell migration, extracellular matrix structure, and protein fusion (**Figure 1A**). KEGG pathway analysis focused on Phagosome、Cytokine-cytokine receptor interaction、Rheumatoid arthritis and ECM-receptor interaction (**Figure 1B**).

### Identification of differentially expressed genes associated with PANoptosis

**Figure 2:**
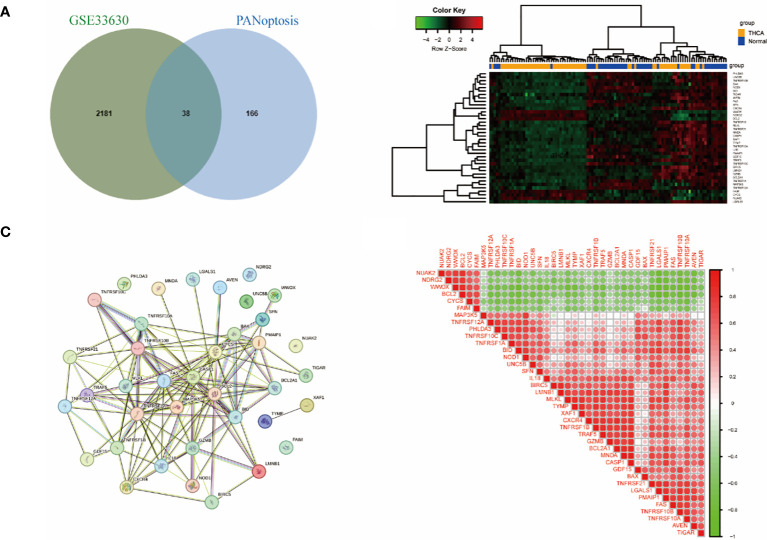
Identification of PANoptosis -related differentially expressed genes in the thyroid cancer training dataset. **(A)** Venn plots of 204 PANoptosis genes and 2216 DEGs. **(B)** Heatmap of the expression of 38 PRGs in the training dataset. Red: low expression level; green: high expression level **(C)** Interaction network graph of PRGs in the STRING database. **(D)** Correlation heatmap of PRGs in the training dataset. THCA, thyroid cancer; DEGs, differentially expressed genes.

A total of 2219 DEGs were obtained in GSE33630 and 204 PANoptosis genes were obtained from GeneCards, and the intersection of the two datasets was taken to obtain 38 PRGs as shown in **Figure 2A**. The heatmap in **Figure 2B** demonstrates the differential expression of the 38 PRGs in GSE33630. We constructed a protein interaction network in which 31 genes had interaction relationships (**Figure 2C**). To explore the links between these genes, we calculated their correlations using the “corrplot” package (**Figure 2D**) and found significant synergistic effects, with most of the genes being significantly positively correlated with each other.

### Constructing functionally enriched networks

**Figure 3:**
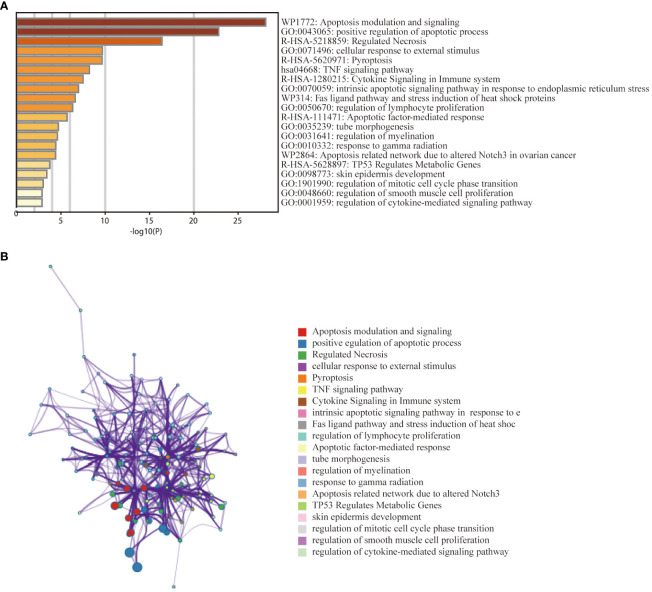
Functional enrichment analysis of PANoptosis -related differentially expressed genes in the thyroid cancer training dataset. **(A)** Bar graph of 20 enriched biological pathways, coloured by p−values. **(B)** Network of enriched terms for specified genes analysed by Metascape, coloured by cluster ID.

To investigate the relationship between PANoptosis genes and the pathogenesis of thyroid cancer, we further explored the functional pathways of PRGs through the metscape database. Functional enrichment analysis showed that the 38 genes were mainly enriched in the positive regulation of apoptosis, pyroptosis, cytokine signalling in the immune system, and regulation of lymphocyte proliferation, etc. (**Figure 3A**). KEGG analysis showed that the genes were mainly related to the TNF signalling pathway. The network graph between the enriched pathways is shown in **Figure 3B**. Nodes with the same pathways tend to cluster together.

### Construction of a PANoptosis risk score model for thyroid cancer

**Figure 4:**
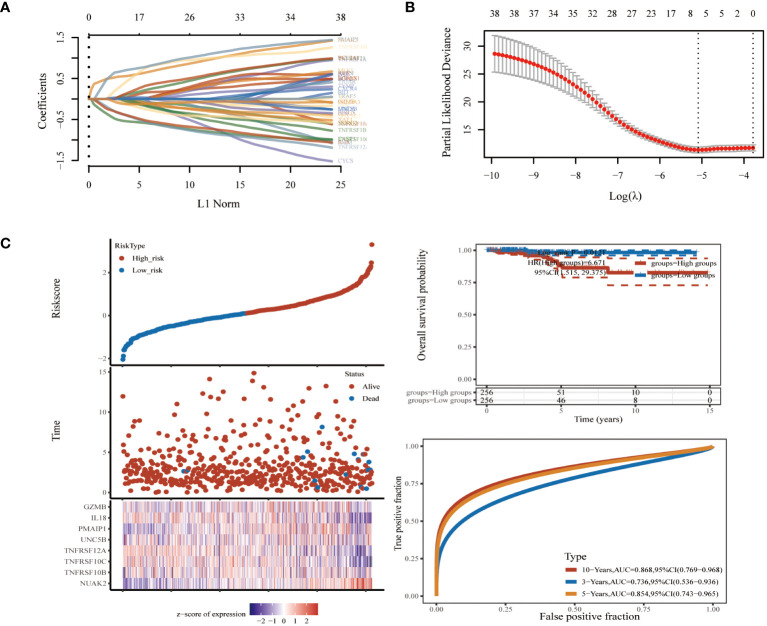
Biomarker identification using LASSO regression based on thyroid cancer dataset in TCGA database. **(A)** Least absolute shrinkage and selection operator (LASSO) regression analysis. **(B)** Cross validation for adjusting parameter selection in LASSO regression. **(C)** Modelling of pan-apoptotic risk score and survival analysis.

**Figure 5:**
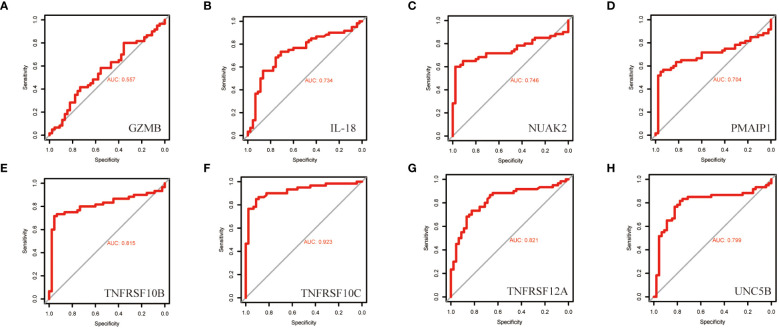
ROC curves of the diagnostic value of the eight biomarkers in the thyroid cancer training dataset. **(A–H)** Subject operating characteristic curves (ROC) of candidate diagnostic markers NUAK2, TNFRSF10B, TNFRSF10C, TNFRSF12A, UNC5B, PMAIP1, IL18 and GZMB in the training dataset.

To further screen PRGs for key genes that play a regulatory role in thyroid cancer disease progression, we constructed a PANoptosis risk score model based on the TCGA database using LASSO regression against 38 PRGs. The LASSO analysis identified eight genes associated with THCA prognosis: NUAK2, TNFRSF10B, TNFRSF10C, TNFRSF12A, UNC5B, PMAIP1, IL18 and GZMB. We established a PANoptosis risk score and survival analysis for THCA as well as the differential expression of these 8 genes in this model (**Figure 4**). We chose the following formula as the risk score formula: lambda.min=0.0062 Riskscore=(0.4977)*NUAK2+(0.0437)*TNFRSF10B+(-0.385)*TNFRSF10C+(-0.2139)*TNFRSF12A+(0.0165)*UNC5B+(0.5133)*PMAIP1+(-0.253)*IL18+(-0.006)*GZMB. We also analysed the ROC curves of this risk model at different times with AUC. the AUC values for 3-year, 5-year and 10-year OS were 0.854, 0.736 and 0.868, respectively (**Figure 4C**). Where the higher AUC value indicates the better predictive ability of the model. In addition, we also calculated the AUC values of these eight candidate genes in the GSE33630 dataset, and all of them, except GZMB, had an AUC greater than 0.7 (**Figures 5A–H**).

### Immune cell infiltrability analysis of the training dataset

**Figure 6:**
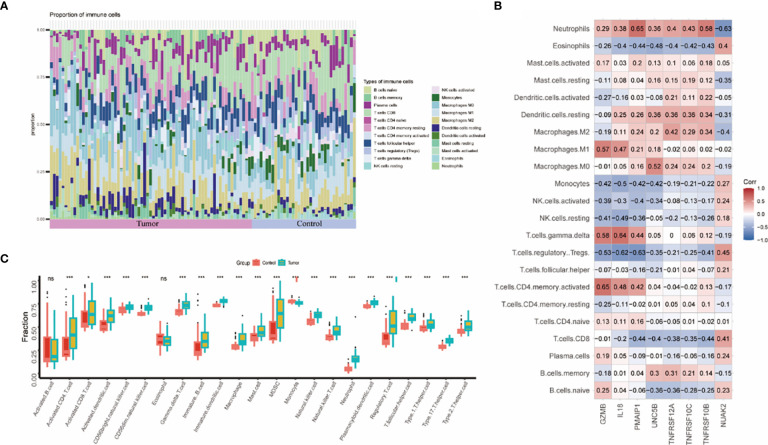
Assessment and visualization of immune cell infiltration in the thyroid cancer training dataset. **(A)** Proportion of immune cell infiltration in the thyroid cancer group and control group. **(B)** Heatmap of 8 biomarkers correlating with immune cells. **(C)** Box line plot of immune cells differentially expressed between thyroid cancer group and control group. (* represented P < 0.05, ** represented P < 0.01 and *** represented P < 0.001).

The immune system of THCA patients plays an important role in disease progression. To investigate the differences in immune cell infiltration between THCA patients and controls, we used the CIBERSORT algorithm. The proportions of immune cells between the two groups are shown in **Figure 6A**. The THCA group showed significantly higher proportions of activated T cells, activated DC cells, naïve B cells, NK cells, and helper T cells compared with the control group (**Figure 6C**). Neutrophils were positively correlated with seven genes except NUAK2. TNFRSF10B, TNFRSF10C, and TNFRSF12A were positively correlated with macrophages, DC cells, and mast cells (**Figure 6B**).

### Construction of hub gene-TF-miRNA transcriptional network

To further explore the potential biological processes of candidate genes in thyroid cancer, we analysed the interactions among candidate genes, transcription factors and miRNAs through the Networkanalyst platform. The TF-gene interaction network was constructed in the ENCODE (https://www.encodeproject.org/) database (**Supplementary Figure S2A**). The TF-miRNA interaction network was obtained in the Regnetwork (http://www.regnetworkweb.org) database (**Supplementary Figure S2B**). The regulatory network was then imported into Cytoscape 3.7.2 for visualization. Combining the regulatory networks revealed that IKZF1 potentially transcriptionally activates PMAIP1, TNFRSF10B and GZMB. The TF-genes network contained 179 TFs, 8 hub genes, and 248 edges. In the TF- miRNA regulatory network, a total of 90 edges and 81 miRNAs interacted with 7 hub genes.

### Validation of candidate diagnostic markers

**Figure 7:**
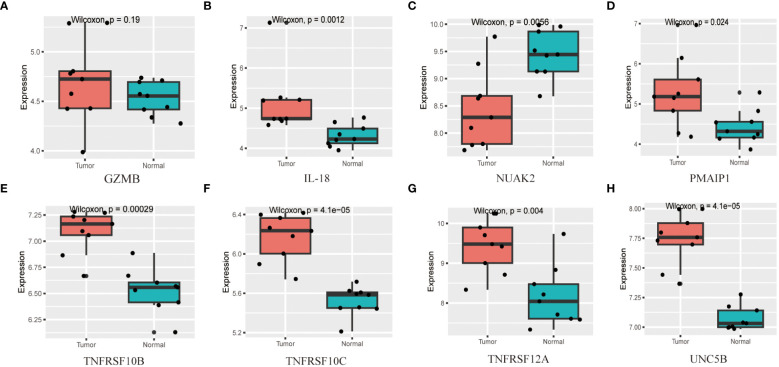
Expression analysis of biomarkers in 8 in the thyroid cancer validation dataset. **(A–H)** Expression levels of candidate diagnostic markers NUAK2, TNFRSF10B, TNFRSF10C, TNFRSF12A, UNC5B, PMAIP1, IL18 and GZMB in the validation dataset.

**Figure 8:**
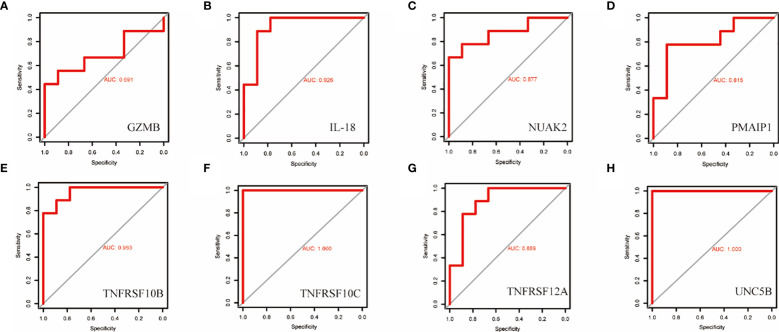
ROC curves for the diagnostic value of the eight biomarkers in the thyroid cancer validation dataset. **(A–H)** Subject operating characteristic curves (ROC) of candidate diagnostic markers NUAK2, TNFRSF10B, TNFRSF10C, TNFRSF12A, UNC5B, PMAIP1, IL18 and GZMB in the validation dataset.

**Figure 9:**
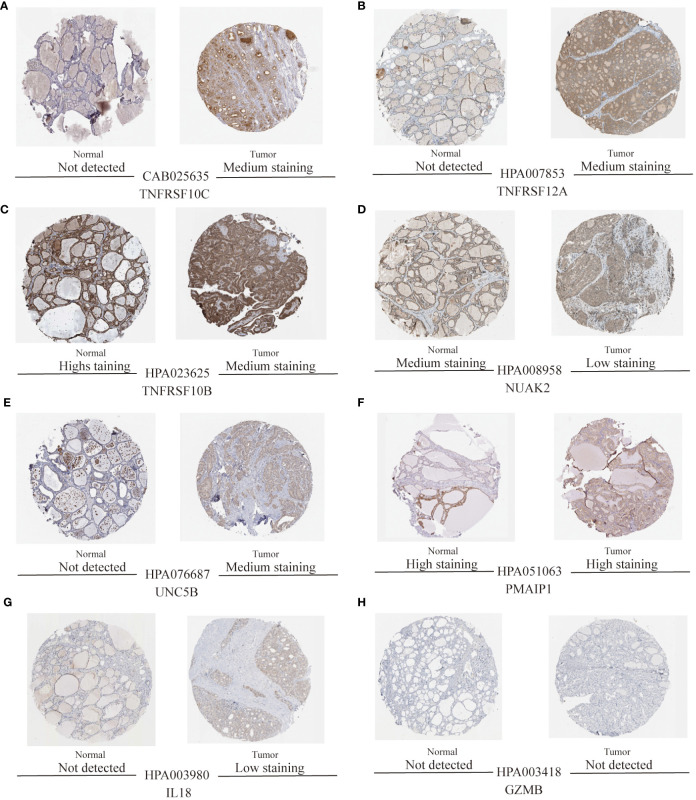
Protein expression levels of eight thyroid cancer biomarkers were analysed based on the HPA database. **(A–H)** Protein expression of candidate diagnostic markers TNFRSF10C, TNFRSF12A, TNFRSF10B, NUAK2, UNC5B, PMAIP1, IL18, and GZMB in thyroid normal and tumour tissues.

To validate the accuracy of the prediction model, we examined the expression of the candidate genes in the validation dataset GSE3467 and ROC analysis. The results showed good diagnostic performance of the predictive model. It is possible that the sample size of the analysis was limited and the difference in the expression of GZMB in the validation dataset was not statistically significant (**Figure 7A**). NUAK2, TNFRSF10B, TNFRSF10C, TNFRSF12A, UNC5B, PMAIP1, and IL18 were expressed in the validation dataset in agreement with the analysis of the assay dataset (**Figures 7B–H**). TNFRSF10B, TNFRSF10C, TNFRSF12A, UNC5B, PMAIP1, and IL18 had increased expression in thyroid cancer tissues, while NUAK2 was decreased. The ROC curves showed that the AUC values of the seven candidate genes were greater than 0.8 except for GZMB (**Figures 8A–H**). Then we verified the protein expression of the candidate genes in the tissues at the protein level. The results showed that except for TNFRSF10B, the expression of other genes was generally consistent with the above analysis. It proved that the PANoptosis prediction model based on 8 key genes was feasible (**Figures 9A–H**).

### Drug-gene interaction prediction

**Figure 10:**
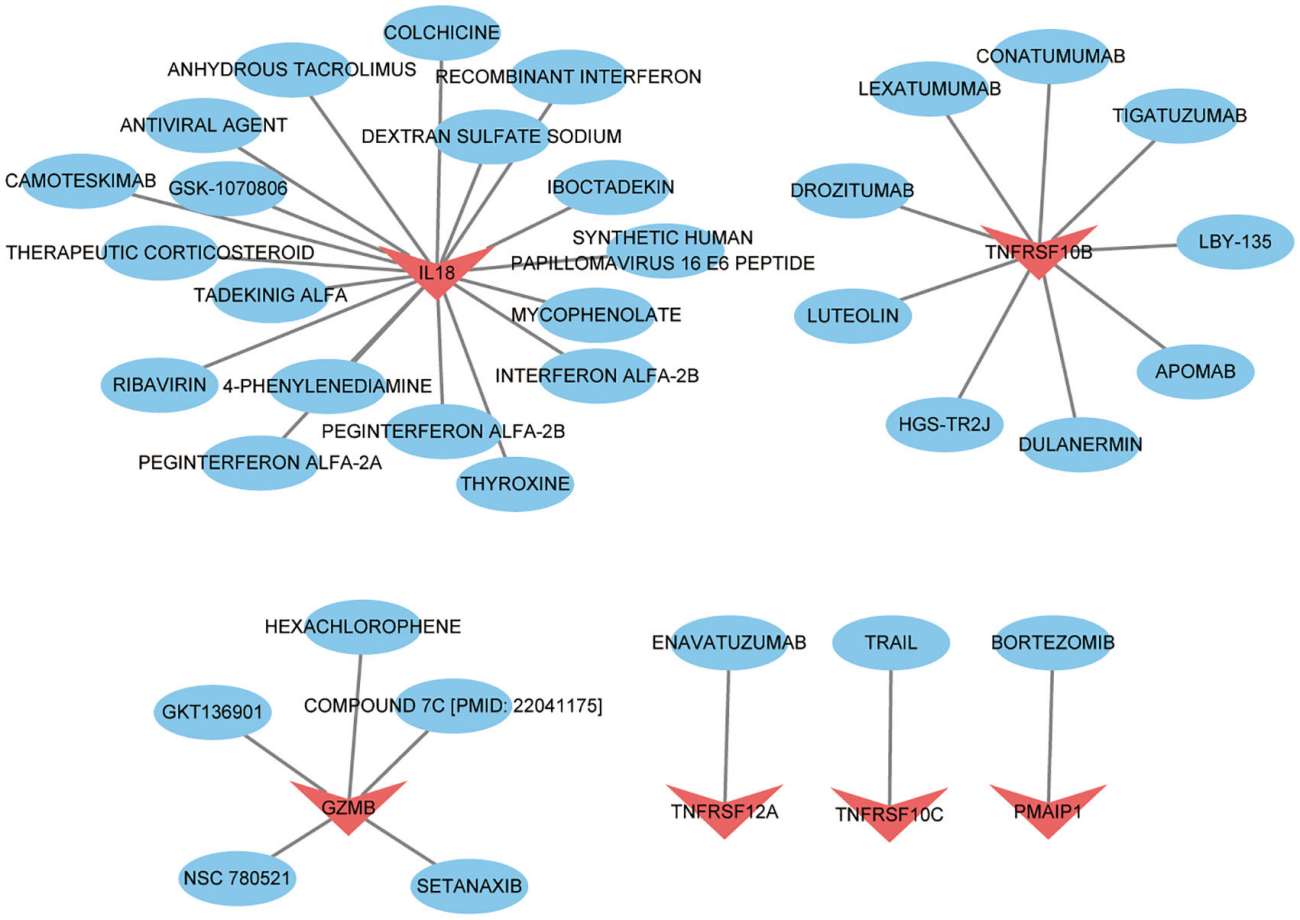
Drug prediction based on key targets in thyroid cancer. Red represents key targets and blue represents predicted drugs.

We imported 8 key targets from the above screening into the DGIbd database for screening small molecule compounds for the treatment of thyroid cancer. In total, 36 drugs interacted with TNFRSF10C, TNFRSF12A, PMAIP1, IL18, GZMB and TNFRSF10B. Visualization was performed using Cytoscape (**Figure 10**). 1 drug targets TNFRSF12A. 1 drug targets PMAIP1. 9 drugs target TNFRSF10B. 19 drugs target IL18. 1 drug targets TNFRSF10C. 5 drugs target GZMB. No potential drugs were identified for UNC5B and NUAK2. Details of these drugs are in **Supplementary Table S1**.

## Discussion

Thyroid cancer has become the most common endocrine system malignancy. The incidence of thyroid cancer is increasing at the highest rate among all malignant tumours. Treatment options for patients with advanced thyroid cancer are very limited. In recent years, with the rapid development of multi-omics research, the understanding of the pathogenesis of different thyroid cancer subtypes has been greatly enhance (22). In particular, the discovery of new thyroid cancer biomarkers has brought hope for the treatment of advanced thyroid cancer. PANoptosis is a novel mode of programmed cell death. PANoptosis has been extensively studied in colorectal, prostate and gastric cancers and some inflammatory diseases (23–27). No study has yet found a link between PANoptosis and thyroid cancer disease progression. The aim of this study was to explore the role of PANoptosis in thyroid cancer disease progression, with the hope of providing new therapeutic targets for the treatment of thyroid cancer.

In this study, a total of 2219 DEGs were identified by analysing the training dataset. In obtained 38 genes that were differentially expressed in thyroid cancer tissues by taking the intersection set. The functional enrichment of the 38 PRGs was mainly related to the regulation of the immune system, with the most significant enrichment in the TNF pathway. We further identified 8 meaningful signature genes using LASSO regression. Then the diagnostic efficacy of these eight genes for thyroid cancer was verified by ROC curves. Among them, the AUC values of TNFRSF10B, TNFRSF10C and TNFRSF12A in the training dataset were all greater than 0.8. TNFRSF10B, TNFRSF10C and TNFRSF12A belong to the tumour necrosis factor receptor superfamily (TNFRSF) which can bind to the tumour necrosis factor superfamily (TNFSF) through the cysteine-rich domains (CRDs). The TNFRSF system contains 19 ligands and 29 receptors, some of which can bind to multiple receptors and regulate complex cellular networks. TNFRSF can assist in the regulation of a variety of cellular functions, including immune responses, inflammatory responses, and cell proliferation, differentiation, and apoptosis (28–31).

In addition, we also analysed the immune cell infiltration in the thyroid cancer group versus the control group in the training dataset. The results showed that activated T cells, NK cells, bone marrow-derived suppressor cells (MDSC), and helper T cells were significantly higher in the tumour group. PANoptosis may be involved in thyroid cancer progression through the immune system. These genes we studied were strongly correlated with mast cell and macrophage infiltration. During the progression of ATC, macrophages shift from the M1 state to the M2 state. The role of macrophages in thyroid cancer progression is worthy of further investigation (2). TNFRSF family-mediated signalling has been a hot topic of research in the field of tumour immunotherapy, with notable findings in CAR-T cell therapy (30, 32, 33). Given that TNFRSF10B, TNFRSF10C and TNFRSF12A are up-regulated in thyroid cancer tissues and correlate with macrophages. They may be able to play an important role in CAR-macrophage therapy in the future. In order to make our study more convincing, the above results were verified again by the validation dataset GSE3467.The expression differences of NUAK2, TNFRSF10B, TNFRSF10C, TNFRSF12A, UNC5B, PMAIP1, and IL18 in the validation dataset were statistically significant. And the AUC values of these genes were all greater than 0.8. The PANoptosis diagnostic model based on 8 key genes performed well in the HPA database, except for TNFRSF10B. Although there are individual differences in the expression of TNFRSF10B, this still proves that our predictive model is feasible.

## Conclusion

our study has some limitations. The results of our analyses were mainly obtained from public databases and lacked sufficient clinical samples for validation. And our study also requires later molecular biology experiments to further explore the hypothesis of the results of this study. However, we finally identified eight PRDEGs as potential targets for thyroid cancer diagnosis and treatment and predicted potential therapeutic agents through this study. The immune microenvironment of thyroid cancer and the link with PRGs were explored by immune infiltration analysis. It gives us a clearer understanding of thyroid cancer and PANoptosis and provides some new ideas for the clinical treatment of thyroid cancer disease.

## Data availability statement

The datasets presented in this study can be found in online repositories. The original contributions presented in the study are included in the article/**Supplementary Material**. Further inquiries can be directed to the corresponding author.

## Author contributions

DW: Writing – review & editing. YL: Writing – original draft.
