# Supplementary material for: Identification of signature genes and immune infiltration analysis in thyroid cancer based on PANoptosis related genes
Source: Front Endocrinol (Lausanne). 2024 Jul 22;15:1397794. doi: 10.3389/fendo.2024.1397794 (PMC11298382; doi:10.3389/fendo.2024.1397794)
Supplement: Supplementary file 1 [file DataSheet_1.docx]

**Identification of signature genes and immune infiltration analysis in thyroid cancer based on PANoptosis related genes**

**Yujie Li^1^Dengqiang Wu^2*^**

The Affiliated Li Huili Hospital, Ningbo University, 1111 Jiangnan Street, Ningbo, Zhejiang Province, China.

^2^ Department of Clinical Laboratory, Ningbo No. 6 Hospital, Ningbo, China.

*Correspondence to: Dengqiang Wu, [wdq19940727@163.com](mailto:wdq19940727@163.com).

Supplementary Figures


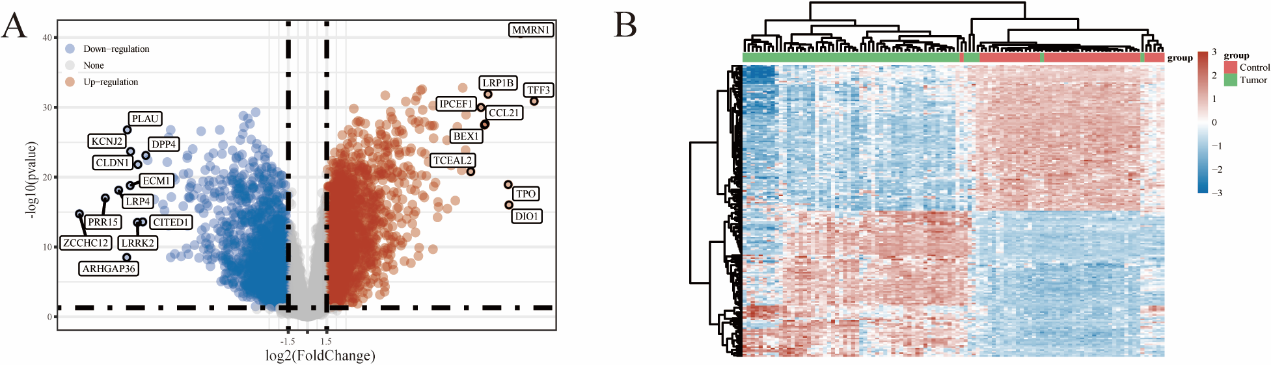


Supplementary Figure1. Identification of differentially expressed genes in the training dataset (A) Volcano plot of differentially expressed genes in the training dataset. Red colour indicates up-regulation and blue colour indicates down-regulation.


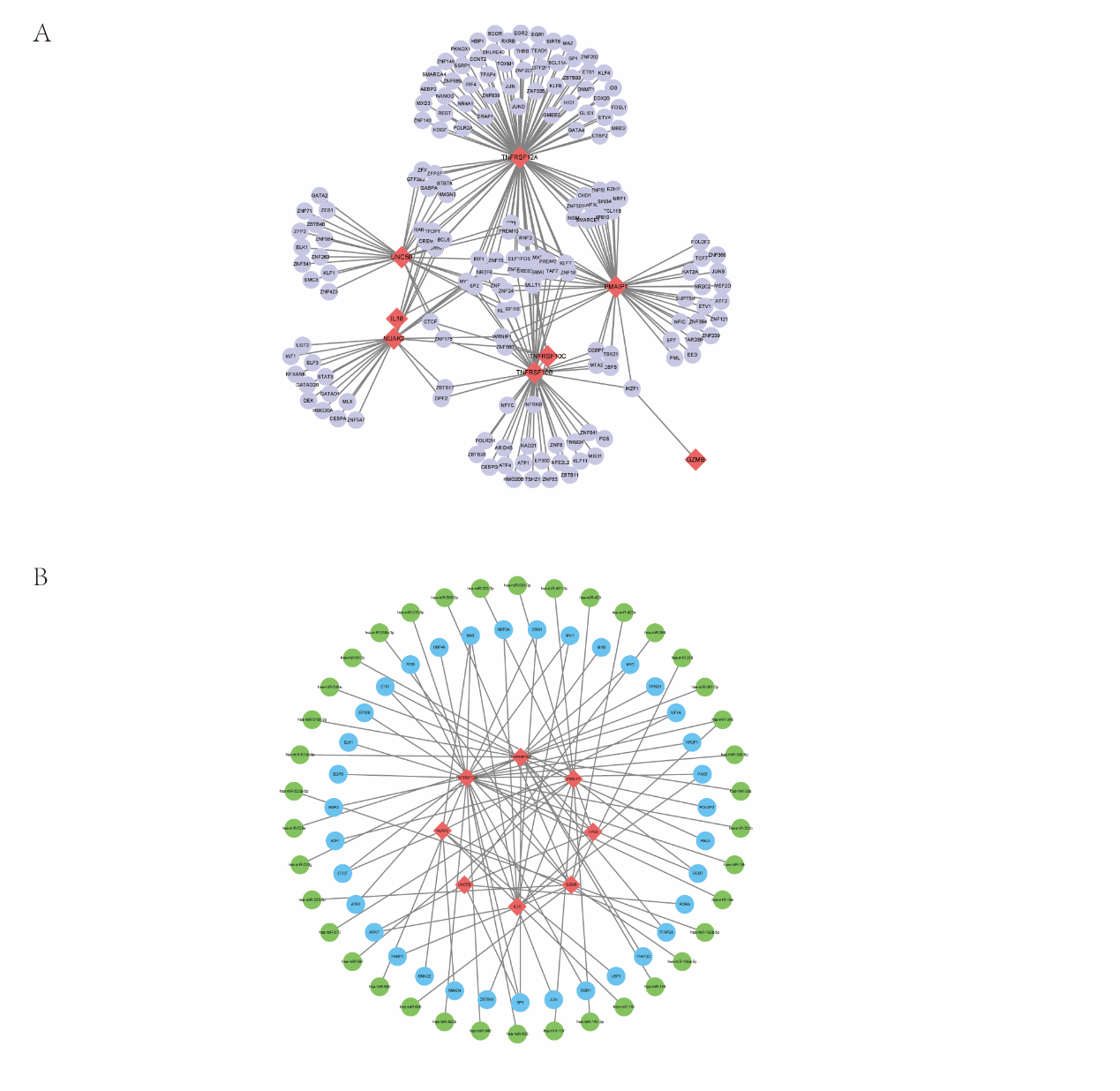


Supplementary Figure2. Hub gene-miRNA-TF regulatory network (A) Transcription factor regulatory network of differentially expressed genes. (B) MiRNA-mRNA-TF regulatory network of differentially expressed genes.

Supplementary Table

| gene | drug | regulatory approval | indication | interaction score |
| --- | --- | --- | --- | --- |
| PMAIP1 | BORTEZOMIB | Approved | antineoplastic agent | 1.221109 |
| IL18 | TADEKINIG ALFA | Not Approved | | 2.763562 |
| IL18 | GSK-1070806 | Not Approved | | 8.290685 |
| IL18 | DEXTRAN SULFATE SODIUM | Not Approved | | 0.69089 |
| IL18 | THERAPEUTIC CORTICOSTEROID | Not Approved | | 0.134808 |
| IL18 | CAMOTESKIMAB | Not Approved | | 2.763562 |
| IL18 | INTERFERON ALFA-2B | Approved | Antineoplastic Agents; Immunomodulatory Agents,for treatment of hepatitis B and C,for treatment of hepatitis,for treatment of hepatitis C | 0.394795 |
| IL18 | IBOCTADEKIN | Not Approved | | 0.921187 |
| IL18 | 4-PHENYLENEDIAMINE | Not Approved | | 0.789589 |
| IL18 | RECOMBINANT INTERFERON | Not Approved | | 0.141721 |
| IL18 | PEGINTERFERON ALFA-2B | Approved |  | 0.172723 |
| IL18 | COLCHICINE | Approved | for treatment of gout | 0.106291 |
| IL18 | ANTIVIRAL AGENT | Not Approved | | 0.614125 |
| IL18 | PEGINTERFERON ALFA-2A | Approved | for treatment of hepatitis B and C,Antineoplastic Agents; Immunomodulatory Agents | 0.19059 |
| IL18 | CAMOTESKIMAB | Not Approved | | 5.527123 |
| IL18 | RIBAVIRIN | Approved |  | 0.145451 |
| IL18 | ANHYDROUS TACROLIMUS | Approved | immunosuppressant | 0.197397 |
| IL18 | THYROXINE | Not Approved | | 0.460594 |
| IL18 | MYCOPHENOLATE | Approved | immunosuppressant | 0.251233 |
| IL18 | SYNTHETIC HUMAN PAPILLOMAVIRUS 16 E6 PEPTIDE | Not Approved | | 5.527123 |
| TNFRSF12A | ENAVATUZUMAB | Not Approved | | 105.0153 |
| TNFRSF10B | DULANERMIN | Not Approved | | 8.751279 |
| TNFRSF10B | LEXATUMUMAB | Not Approved | antineoplastic agent | 29.17093 |
| TNFRSF10B | LUTEOLIN | Not Approved | | 0.364637 |
| TNFRSF10B | DROZITUMAB | Not Approved | | 11.66837 |
| TNFRSF10B | APOMAB | Not Approved | | 11.66837 |
| TNFRSF10B | LBY-135 | Not Approved | | 17.50256 |
| TNFRSF10B | CONATUMUMAB | Not Approved | | 5.834186 |
| TNFRSF10B | TIGATUZUMAB | Not Approved | | 5.834186 |
| TNFRSF10B | HGS-TR2J | Not Approved | antineoplastic agent | 5.834186 |
| TNFRSF10C | TRAIL | Not Approved | | 10.50153 |
| GZMB | GKT136901 | Not Approved | | 3.500511 |
| GZMB | COMPOUND 7C [PMID: 22041175] | Not Approved | | 2.625384 |
| GZMB | HEXACHLOROPHENE | Approved |  | 0.51227 |
| GZMB | NSC 780521 | Not Approved | | 2.625384 |
| GZMB | SETANAXIB | Not Approved | | 2.625384 |
